# Biology of HDL: From Structural Heterogeneity to Dysfunctional Remodeling in Cardiovascular Disease and Comorbidities

**DOI:** 10.3390/antiox15070864

**Published:** 2026-07-10

**Authors:** Yihang Cai, Kehan Li, Huibo Ma, Jianqiang Wu, Yuehong Zheng

**Affiliations:** 1 Department of Vascular Surgery, State Key Laboratory of Complex Severe and Rare Disease, Peking Union Medical College Hospital, Chinese Academy of Medical Sciences and Peking Union Medical College, Beijing 100730, China; b2025001046@student.pumc.edu.cn (Y.C.); 48upvobr@jiouba.cn (K.L.); b2024001242@student.pumc.edu.cn (H.M.); 2 National Infrastructure for Translational Medicine, Institute of Clinical Medicine, Center for Biomarker Discovery and Validation, Peking Union Medical College Hospital, Chinese Academy of Medical Sciences and Peking Union Medical College, Beijing 100730, China

**Keywords:** cardiovascular diseases, HDL, dysfunctional HDL, diabetes, proteomics, lipidomics, noncoding RNA, cholesterol efflux capacity

## Abstract

The pathogenesis of cardiovascular diseases (CVDs) is intimately linked to cholesterol dysregulation. While high-density lipoprotein cholesterol (HDL-C) is classically considered cardioprotective, contemporary epidemiological evidence reveals a noncausal, often U-shaped, relationship with CVD risk. Static measurements of HDL-C obscure the structural and functional heterogeneity of circulating HDL particles. Under pathological stress, HDL undergoes extensive structural remodeling into dysfunctional HDL, thereby losing its vasculoprotective properties and instead mediating proatherogenic and proinflammatory responses. This review critically evaluates the biogenesis, maturation, and metabolic trajectory of HDL. By integrating recent advancements in proteomics and lipidomics, we map the intricate compositional shifts within HDL subpopulations and clarify the regulatory roles of HDL-associated microRNAs in intercellular communication. We investigate the specific drivers of HDL dysfunction, which is often exacerbated by comorbidities such as diabetes and chronic kidney disease. Furthermore, we outline the methodological transition from automated homogeneous HDL-C quantification to multidimensional profiling. Shifting the clinical focus from HDL quantity to functional quality resolves the HDL-C paradox, helping to drive the development of precision lipidology and targeted therapies to reverse HDL dysfunction in CVDs.

## 1. Introduction

Cardiovascular diseases (CVDs) are among the leading causes of death and disability worldwide. Notably, over the three decades spanning the 1990s to 2019, global epidemiological data indicate that the incidence of CVDs increased from 271 million cases to 523 million cases, while the associated mortality rate increased correspondingly from 12.1 million deaths to 18.6 million deaths [1]. From 2025 to 2050, the global crude incidence of CVD is projected to soar from 598 million cases to 1.14 billion cases, while the crude mortality rate is expected to increase from 20.5 million cases to 35.6 million cases. Although age-standardized mortality rates are likely to decline, population growth and aging will lead to a significant increase in the absolute number of cases and deaths from CVDs [2]. Among the multiple risk factors contributing to CVD pathogenesis, dysregulated cholesterol homeostasis is a critical determinant. Esko Nikkilä pioneered scientific inquiry into high-density lipoprotein cholesterol (HDL-C) in 1953, when he reported significantly lower HDL-C levels in patients with atherosclerotic cardiovascular disease (ASCVD) than in healthy controls [3]. Throughout the 1950s and 1960s, this observation was rigorously validated by subsequent case-control studies [4,5,6,7,8]. This negative correlation was later confirmed in general populations, such as in the 1976 cross-sectional study of Japanese-American men in Hawaii and received definitive support from four major prospective studies in the United States by the late 1980s [9,10]. Consequently, HDL-C has been heralded as good cholesterol because of its consistent inverse clinical association with ASCVD risk [11,12]. However, recent evidence has challenged this linear paradigm. Mendelian randomization studies have failed to establish a causal link between HDL-C levels and CVD risk, and pharmacological interventions aimed at elevating HDL-C have largely been disappointing in clinical trials [13,14]. Emerging data suggest a U-shaped relationship, in which both excessively low and high HDL-C levels are associated with increased cardiovascular risk [15,16]. This discrepancy highlights that the HDL-C concentration does not fully capture the complex biological roles of HDL. In the pathological environment of CVDs, HDL undergoes significant structural and functional remodeling [17]. The advent of high-resolution omics technologies has revealed that alterations in HDL-associated proteins, lipids, and noncoding RNAs can transform HDL from a vasoprotective agent into a dysfunctional or even proinflammatory particle. This review systematically summarizes HDL structure and function, evaluates contemporary isolation and assessment methodologies, and explores how newly identified heterogeneous components contribute to CVD pathogenesis.

## 2. The Architecture and Lifecycle of HDL

### 2.1. Structural Complexity and Heterogeneity of HDL

**Figure 1:**
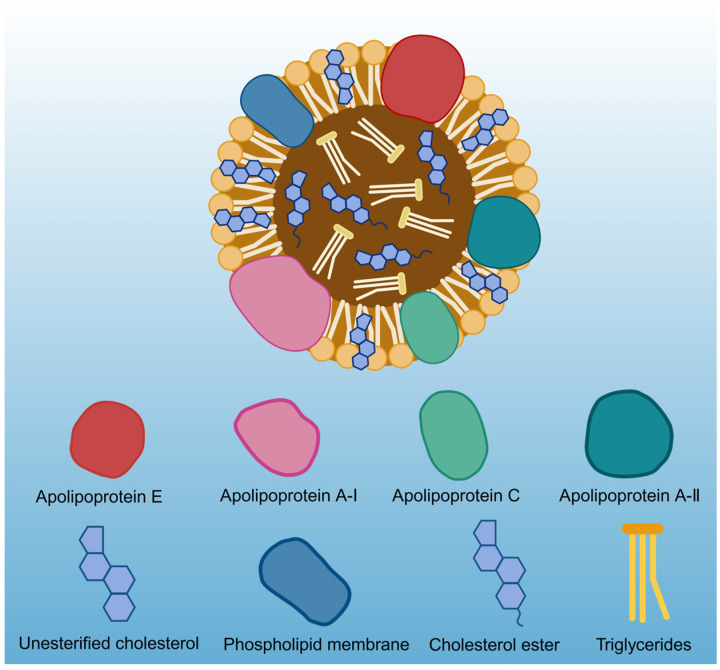
Schematic illustration of the structural architecture and molecular composition of a mature HDL particle. Created in BioRender. serwe, F. (2026) https://BioRender.com/nbrd1bx.

HDL is the smallest and densest class of lipoprotein and has a unique proteomodified lipid structure. The HDL particle structure is hydrophobic, consisting of cholesteryl esters (CEs) and a small number of triglycerides (TGs). The core of HDL is surrounded by a polar monolayer consisting of phospholipids, free cholesterol, and various apolipoproteins (Figure 1). Apolipoprotein A-I (ApoA-I) is approximately 70% of the total HDL protein mass and is necessary for structural integrity and biological functions [18]. The amphipathic α-helical structure wraps around the lipid bilayer and shows that ApoA-I not only functions as a structural scaffold of HDL but also plays a role in activating metabolic enzymes such as lecithin cholesterol acyltransferase (LCAT) and in mediating HDL interactions with cell surface receptors [19,20]. The structural scaffold of a diverse proteome and lipidome coordinates interactions that define size, charge, and functionality [21].

### 2.2. Biogenesis and Maturation of HDL

ApoA-I is the primary structural protein of HDL. It is synthesized and secreted by the liver and intestine to initiate nascent HDL formation via interaction with ATP-binding cassette transporter A1 (ABCA1). ABCA1 facilitates the efflux of cellular phospholipids and free cholesterol into ApoA-I and forms discoidal, nascent HDL particles [22]. LCAT mediates the maturation of these nascent disks by converting surface-free cholesterol into hydrophobic CEs, which partition into the particle core and drive the transition into mature spherical HDL.

### 2.3. Remodeling in Circulation and Reverse Cholesterol Transport

**Figure 2:**
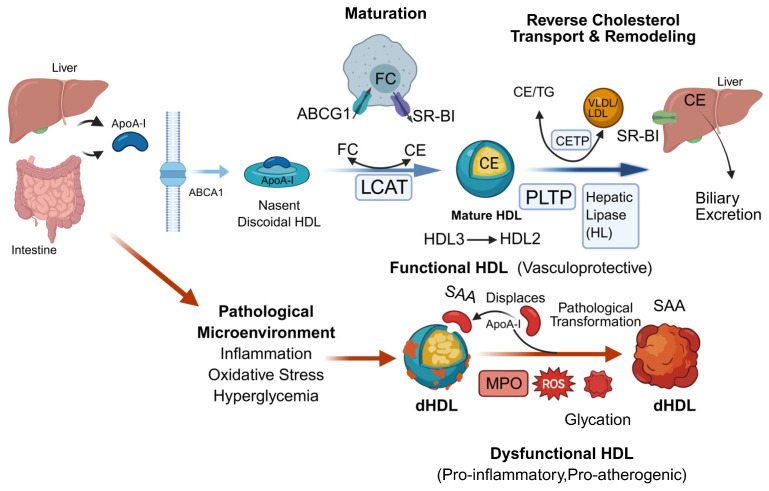
Lifecycle of HDL and pathological transformation into dysfunctional HDL. ApoA-I secreted by the liver and intestine forms nascent discoidal HDL via ABCA1-mediated phospholipid and free cholesterol efflux. LCAT converts free cholesterol into cholesteryl esters, maturing discoidal HDL into spherical HDL (HDL3 to HDL2), with continuous cholesterol uptake via ABCG1 and SR-BI. CETP, PLTP, and hepatic lipase mediate HDL remodeling in the circulation, and mature HDL facilitates reverse cholesterol transport through hepatic SR-BI-mediated CE uptake and subsequent biliary excretion, exerting vasculoprotective effects. In inflammatory, oxidative, and hyperglycemic microenvironments, functional HDL is remodeled into dysfunctional HDL through SAA-mediated ApoA-I displacement, MPO/ROS-induced oxidative modification, and nonenzymatic glycation, resulting in proinflammatory and proatherogenic particles. Abbreviations: FC, free cholesterol; CE, cholesteryl esters; TG, triglyceride; ABCG1, ATP-binding cassette transporter G1; SR-BI, scavenger receptor class B type I; PLTP, phospholipid-transfer protein; LCAT, lecithin cholesterol acyltransferase; HL, hepatic lipase; MPO, myeloperoxidase; ROS, reactive oxygen species; SAA, serum amyloid A. Created in BioRender. serwe, F. (2026) https://BioRender.com/ywhmw5m.

Upon initial maturation, HDL undergoes dynamic remodeling by key enzymes and transfer proteins, which determines its metabolism. Cholesteryl ester-transfer protein (CETP) transfers CEs from HDL to very-low-density lipoproteins and low-density lipoproteins (LDLs) in exchange for triglycerides, altering the atherogenic properties of these lipoprotein particles [23]. This process is complemented by the activity of phospholipid-transfer protein (PLTP), which modulates particle size through the interlipoprotein transfer of phospholipids and subsequent generation of lipid-poor preβ-HDL. Furthermore, the structural integrity of HDL is regulated by hepatic lipase and endothelial lipase. These lipases facilitate the hydrolysis of HDL-associated phospholipids and triglycerides, producing smaller, denser particles and possibly dissociating ApoA-I for renal clearance [24]. The final stage of the HDL lifecycle is the delivery of cholesterol to the liver via reverse cholesterol transport (RCT). RCT occurs through two primary pathways. In the direct pathway, scavenger receptor class B type I (SR-BI) selectively uptakes cholesterol esters into hepatocytes without requiring internalization of entire HDL particles. Alternatively, the indirect pathway involves the CETP-mediated transfer of cholesterol to apoB-containing lipoproteins, which are subsequently cleared from the circulation via LDL receptor-mediated endocytosis [22,25] (Figure 2).

## 3. Functions of HDL

### 3.1. The RCT Pathway

The best-characterized atheroprotective feature of HDL is its ability to facilitate cholesterol efflux from peripheral cells, particularly macrophages [26]. In fact, this function is the rate-limiting step of RCT, as extrahepatic tissues lack the enzymatic machinery to catabolize sterol rings. In vascular tissue, the sequestration of intracellular cholesterol, mostly from modified LDL, drives the transition of macrophages into foam cells. As the hallmark of early atherogenesis, the formation of foam cells reflects a breakdown in lipid trafficking. HDL is the primary mechanism for mobilizing this excess sterol burden, maintaining vascular lipid homeostasis, and inhibiting plaque formation. Unlike systemic HDL-C levels, cholesterol efflux capacity (CEC) measures how efficiently HDL and its lipid-poor ApoA-I precursor extract cholesterol from macrophages via ABCA1 and ATP-binding cassette transporter G1 (ABCG1) transporters [27,28]. Moreover, continuous esterification of cholesterol by LCAT and subsequent remodeling by CETP and PLTP create a concentration gradient that promotes sustained lipid flux from the periphery to the liver. Finally, SR-BI mediates HDL-C uptake by hepatocytes, and once internalized, HDL-C is recycled or converted into bile acids for fecal excretion [25]. This reduces the total body cholesterol burden. Recent studies have shown that efflux efficiency is a better predictor of cardiovascular risk than the static concentration of HDL-C [29]. This function is often compromised in patients with metabolic syndrome or chronic inflammation, where dysfunctional HDL fails to clear cholesterol from the subendothelium.

### 3.2. Antioxidant Effects of HDL

HDL serves not only a cholesterol transport function but also acts as an antioxidant, preventing oxidized low-density lipoproteins (oxLDLs) from exerting pro-atherogenic effects [30]. HDL achieves this goal by capturing and neutralizing lipid hydroperoxides derived from both circulating LDLs and damaged cell membranes. HDL-associated enzymes, including paraoxonase 1 (PON1) and platelet-activating factor acetylhydrolase (PAF-AH), catalyze the hydrolysis of oxidized phospholipids, thereby interrupting lipid peroxidation cascades and playing a pivotal role in mitigating oxidative stress [31,32]. PON1 catalyzes the hydrolysis of lipid peroxides and lactones, thereby preventing the oxidative modification of LDL and protecting the endothelium from oxLDL-induced injury. PAF-AH hydrolyzes oxidized fatty acid compounds in phospholipids, neutralizing bioactive truncated lipids that would otherwise trigger inflammatory cascades. In CVD-related myocardial injury, mitochondrial dysfunction is a primary source of reactive oxygen species (ROS) [33]. ApoA-I, the core component of HDL, is the key effector of resistance to oxidative stress [34]. ApoA-I and HDL-bound sphingosine-1-phosphate (S1P) act as signaling ligands that stabilize mitochondrial membranes and attenuate ROS production to prevent mitochondrial permeability transition pore opening and subsequent apoptosis [35]. HDL antioxidant activity depends heavily on its subfraction distribution and apolipoprotein composition. Small dense HDL particles are more efficient than large buoyant subfractions because their surface concentration is higher and their spatial interactions with LDL particles are more favorable [36].

### 3.3. Mechanisms of HDL-Mediated Anti-Inflammation

HDL exhibits remarkable functional plasticity in its immunological profile, dynamically adapts to physiological or pathological environments, and acts as a powerful anti-inflammatory agent under steady-state conditions. As a regulator of endothelial homeostasis, HDL exerts its most direct anti-inflammatory action by inhibiting leukocyte adhesion and invasion into the vascular wall. Together with ApoA-I, HDL inhibits the expression of CD11b on neutrophil and monocyte surfaces, thereby attenuating the activation of these immune cells and their adhesion to endothelial cells. Additionally, HDL potently suppresses the inflammatory cascade by inhibiting nuclear factor kappa B (NF-κB) signaling in endothelial cells. This inhibition subsequently reduces the expression of critical adhesion molecules, including vascular cell adhesion molecule-1, intercellular adhesion molecule-1, and E-selectin, as well as chemokines such as monocyte chemoattractant protein-1. These effects collectively prevent the transendothelial migration and subendothelial infiltration of monocytes and neutrophils [37,38]. Macrophages represent key regulatory targets of HDL. Initially, cholesterol-rich microdomains, known as lipid rafts, on the macrophage membrane serve as signaling platforms for pattern recognition receptors such as Toll-like receptor 4. HDL mediates cholesterol efflux via its receptors ABCA1 and ABCG1, depleting cholesterol in lipid rafts and causing structural disruption. This impairs the aggregation of Toll-like receptor 4 and the downstream MyD88/NF-κB signaling, reducing the production of proinflammatory cytokines such as tumor necrosis factor-alpha (TNF-α) and interleukin-6 (IL-6), a mechanism that directly links cholesterol metabolism to inflammatory signaling [39]. Furthermore, HDL induces macrophages to express transcriptional repressors such as activating transcription factor 3, thereby globally suppressing the expression of proinflammatory genes [40]. HDL exerts systemic control over inflammation by regulating the proliferation of hematopoietic stem and progenitor cells. This reduces the number of proinflammatory monocytes and neutrophils in the systemic circulation, alleviating the systemic chronic inflammatory burden at its source and preventing an overabundance of inflammatory cells from being recruited to vascular lesion sites [41,42,43]. Through enzymatic detoxification, PON1 and PAF-AH on HDL particles hydrolyze and inactivate proinflammatory lipid mediators such as oxidized phospholipids and platelet-activating factors. Notably, PON1 requires a stable association with HDL for full enzymatic and anti-inflammatory activity [44]. Furthermore, HDL particles are rich in proteases and endogenous protease inhibitors, including α-1 antitrypsin, thereby functioning as a platform for spatially controlled proteolytic regulation. For example, HDL-bound α-1 antitrypsin can be efficiently transported to inflammatory sites to inhibit the destructive effects of proteases such as neutrophil elastase [45]. HDL also regulates the cascades of the complement and coagulation/fibrinolysis systems, preventing excessive complement activation that generates anaphylatoxins such as C3a and C5a and maintaining the balance between thrombosis and thrombolysis [46]. HDL delivers sustained protection by inducing expression of the specific anti-inflammatory protein 3-β-hydroxysteroid-Δ24 reductase (DHCR24, also known as seladin-1) in endothelial cells. Its inhibitory effect on TNF-α-induced adhesion molecule expression persists for hours or even 24 h after HDL is removed from the cell culture system. DHCR24 not only participates in cholesterol synthesis but also has antioxidant activity and suppresses the NF-κB pathway. This endows endothelial cells with long-lasting anti-inflammatory properties [43].

### 3.4. HDL in Thrombosis and Fibrinolysis

Thrombosis is a pathological event in the progression of atherosclerosis and acute coronary syndrome. This process is driven by an interplay between platelet hyperactivation, endothelial dysfunction, and an imbalance between the coagulation and fibrinolytic systems. HDL exerts multifaceted antithrombotic effects that prevent both arterial thrombosis and venous thrombosis. The antithrombotic capacity of HDL is primarily rooted in its interaction with endothelial SR-BI. The mechanism involves HDL binding to SR-BI on the surface of endothelial cells, thereby activating signaling pathways such as the phosphoinositide 3-kinase (PI3K)/protein kinase B (Akt) and mitogen-activated protein kinase (MAPK) pathways [47]. This leads to enhanced endothelial nitric oxide synthase (eNOS) expression and activity, thereby increasing nitric oxide (NO) production and preserving a membrane lipid environment conducive to eNOS function [48]. The resulting increase in NO promotes vasodilation, inhibits platelet aggregation, and has anti-inflammatory effects. Furthermore, HDL and S1P activate the Akt survival pathway via the receptors SR-BI and S1PR1/3, thereby inhibiting endothelial cell apoptosis, reducing exposure of procoagulant phosphatidylserine, and decreasing the release of procoagulant particles [49,50]. By accelerating reendothelialization and cellular migration, HDL ensures that the subendothelial matrix remains sequestered from circulating procoagulant factors [51]. HDL also inhibits activation of the coagulation cascade by targeting several critical steps. Tissue factor (TF) expression is downregulated by reconstituted HDL in thrombin-stimulated endothelial cells. This effect involves inhibition of the small GTPase RhoA and activation of PI3K signaling [52]. Moreover, HDL, especially the HDL2 subclass, potentiates the anticoagulant activity of activated protein C (APC) and its cofactor protein S. APC inhibits thrombin generation by inactivating factors Va and VIIIa [53]. Specific HDL-associated glycosphingolipids, such as glucosylceramide, act as lipid cofactors for APC, enhancing its function; reduced plasma levels of glucosylceramide are associated with increased venous thrombosis risk [54,55]. HDL also contains sphingosine, which directly suppresses the prothrombinase complex and reduces thrombin formation [56]. Moreover, HDL-associated apolipoproteins, including ApoA-IV, bind to platelet integrin αIIbβ3 and inhibit its interaction with fibrinogen, disrupting thrombin-mediated platelet aggregation and fibrin clot formation. Platelet activation is central to arterial thrombosis. The inhibition of platelet activation by HDL involves both indirect endothelial-dependent and direct intracellular pathways. By stimulating endothelial PGI2 and NO synthesis, HDL increases intraplatelet cyclic adenosine monophosphate (cAMP) and cyclic guanosine monophosphate (cGMP) levels, thereby raising the activation threshold. Specifically, HDL3 interferes with thrombin-induced phosphoinositide signaling, reducing the production of 1,2-diacylglycerol and inositol 1,4,5-triphosphate [57]. Structurally, ApoA-I obstructs the interaction between agonists such as ADP and their respective platelet receptors [58]. Conversely, the transition to dysfunctional ox-HDL abrogates its protective effects, leading to the upregulation of endothelial plasminogen activator inhibitor-1 (PAI-1) activity and the suppression of fibrinolytic activity. In addition, HDL-associated proteins, including ApoA-I and clusterin, inhibit the formation of the complement membrane attack complex, conferring protection against endothelial injury through excessive complement activation.

## 4. Progress in Multiomics Research on HDL

Significant progress has been made in omics research in recent years. High-resolution proteomics and lipidomics technologies enable comprehensive quantification of proteins and lipids in clinical samples. These methods can characterize the molecular profiles of lipoproteins and HDL subpopulations and reveal the proteome–lipidome connectivity of lipoproteins and HDL particles. Omics studies have not only facilitated the discovery of new protein fractions in HDL, such as those involved in the acute phase response, complement activation, the immune response, the inflammatory response, and protease inhibition, but have also demonstrated a wide range of clinical applications. Multiomics has become the first choice for screening new biomarkers and therapeutic targets for CVD.

### 4.1. Progress in Proteomic Studies of HDL

Accumulating clinical evidence indicates that therapeutic strategies aimed at elevating HDL-C levels do not reduce cardiovascular risk [59]. This may be due to the intricate structural diversity of HDL particles, which leads to unintended drug effects. The heterogeneous nature of HDL particles is characterized by variations in their lipid–protein composition, resulting in a diverse array of subclasses with varying physiological roles. Comprehensive proteomic profiling has revealed 16 distinct HDL subspecies. Beyond its canonical role in lipid metabolism, the HDL proteome orchestrates a broad spectrum of systemic processes, including modulation of the acute-phase response, complement regulation, protease inhibition, hemostasis, antioxidant defense, and innate immunity [60], as summarized in Table 1.

The transition of HDL from a vasculoprotective agent into a pro-pathogenic mediator is thought to be partly driven by proteomic remodeling within the pathological environment. To understand this dynamic process, stable isotope-based mass spectrometry was used to quantify the in vivo kinetics of HDL metabolism and RCT. This approach shows the heterogeneity of HDL and its protein components, demonstrating their precise control over velocity and biological activities. Assessing HDL from a metabolic function perspective offers a novel pathway for investigation [61]. Label-free quantitative proteomics has enabled high-resolution, independent estimation of the proteomic profiles of functionally distinct HDL subpopulations. Function is determined not only by size but also by the identity and relative abundance of associated apolipoproteins. Two independent cohorts have validated these associations: ApoA-II positively predicts preβ-1 HDL cholesterol efflux capacity, while ApoE inversely correlates with α-1/α-2 HDL function in overweight/obese patients with coronary artery disease (CAD) and in healthy young European males [62,63]. Collectively, these results suggest that specific apolipoprotein fractions may serve as robust, mechanistically informed biomarkers of HDL subset functionality [63]. In CVDs, the proteomic composition of HDL often changes, leading to alterations in homeostasis, acute-phase reactants, and prothrombotic factors. High-resolution mass spectrometry revealed that ApoA-I and PON1 levels in HDL particles were significantly reduced in patients with acute coronary syndrome or vulnerable plaques, thereby impairing RCT capacity and antioxidant activity. Simultaneously, these remodeled particles are enriched with serum amyloid A (SAA) and ceruloplasmin [64]. This proteomic change, particularly the SAA/ApoA-I ratio, can be used to identify high-risk, rupture-prone plaques that are not easily detected by luminal stenosis alone.

**Table 1:** Core functional categories of HDL.

Functional Category	Protein Species	Biological Mechanisms	Clinical Significance	Ref
Lipid Metabolism & Transport	ApoA-I, ApoE, LCAT, CETP, PLTP	Mediates macrophage cholesterol efflux, RCT, HDL maturation and lipidation, hepatic cholesterol clearance	Reduced efflux capacity is a hallmark of atherosclerosis and CVD risk	[43]
Acute-Phase Response & Inflammation	SAA1/2/4, ApoJ (Clusterin), Haptoglobin	Modulates inflammatory signaling cascades; buffers pro-inflammatory cytokine activity; SAA enrichment displaces ApoA-I	Conversion of anti-inflammatory HDL into pro-inflammatory dysfunctional HDL (dHDL)	[64]
Complement Regulation	Complement C3, C4A/B, C9, Factor H	Regulates the activation of complement cascade, restricts excessive complement activation in the vascular wall, prevents endothelial damage	Dysregulation leads to increased leukocyte recruitment and plaque instability	[65]
Protease Inhibition	α-1-antitrypsin, Serpin family	Inhibits elastase and matrix metalloproteinases, protects the integrity of extracellular matrix, prevents excessive vascular wall remodeling	Deficiency promotes extracellular matrix degradation and vascular remodeling	[46]
Hemostasis & Coagulation	Kininogen-1, Fibrinogen, Plasminogen	Modulates platelet activation, coagulation cascade and fibrinolysis, maintains vascular antithrombotic homeostasis	Pro-thrombotic protein enrichment in dHDL enhances cardiovascular events	[12]
Antioxidant Activity	PON1, PON3, PAF-AH, GPx3	Scavenges ROS, neutralizes lipid hydroperoxides and prevents LDL and HDL oxidation, preserves endothelial NO bioavailability	Loss of PON1 activity is a key driver of oxidative stress in the arterial wall	[44]
Innate Immunity & Metal Homeostasis	ApoL1, Ceruloplasmin, Transferrin, Lipocalin-2	Provides antimicrobial defense and sequesters transition metal ions, regulates innate immune cell activation in the vascular wall	Prevents Fenton-reaction-mediated oxidative damage and infection	[17]

HDL remodeling is ethnically and genetically specific, restricting certain biomarkers to distinct populations. Label-free quantitative proteomics identified a unique cardiotoxic profile in a high-risk South Asian population, with decreased ApoA-IV and ApoF alongside elevated ApoC-III [62]. Large-scale, multiethnic data from the Dallas Heart Study demonstrated that APOL1 risk variants are uniquely confined to cohorts of African descent [66,67].

A major advancement in HDL proteomics is the study of its regulatory role in the complement and coagulation systems [46]. In patients with coronary heart disease, HDL subspecies that are typically associated with complement inhibition, such as those containing Clusterin or Complement C3, often exhibit dysfunction [68]. Moreover, the enrichment of ApoC-III and fibrinogen-like protein 1 within HDL particles in CVDs has been shown to promote leukocyte adhesion and may activate procoagulant pathways, thereby counteracting the physiological role of HDL in maintaining hemostatic equilibrium [69,70]. Dysfunctional HDL subfractions may fail to neutralize the alternative complement pathway, potentially accelerating endothelial damage and the recruitment of inflammatory cells to the subendothelial space. The clinical application of HDL proteomics transcends mere risk stratification. Characterization of the 16 previously identified subspecies can reveal specific pathological clusters that may serve as new therapeutic targets. For example, therapeutic approaches that aim to selectively deplete ApoC-III-enriched HDL or restore PON1 activity may represent a move toward precision medicine in lipidology [71,72].

### 4.2. Advances in Lipidomics of HDL

Plasma lipids have been used to predict and prevent CVDs. Advancements in lipidomics have enabled researchers to thoroughly investigate the metabolic dysregulation associated with CVDs and their genetic determinants. The lipidome is the comprehensive lipid content in a cell or tissue, and it has been divided into eight classes by the LIPID MAPS consortium: fatty acyls, glycerolipids, glycerophospholipids, sphingolipids, sterols, prenyl alcohols, glycolipids, and polyketides [73]. HDL contains various classes of phospholipids, sphingolipids, and glycerolipids, and two complementary analytical tools are being employed to characterize its lipidome [74]. Nuclear magnetic resonance (NMR) enables rapid, high-throughput profiling of major lipid classes and lipoprotein particle size distributions in HDL but provides only limited resolution for low-abundance lipid species and structural isomers [75]. In contrast, liquid chromatography-tandem mass spectrometry (LC-MS/MS) provides sensitivity, specificity, and coverage of rare lipid species such as plasmalogens, S1P, and ceramides, enabling precise quantification of individual molecular species that govern HDL function [76]. These techniques are used to define functional lipid signatures beyond standard lipid measurements.

Phosphatidylcholine is the most abundant lipid in HDL, followed by CEs, as demonstrated by density gradient ultracentrifugation and NMR spectroscopy [77,78]. Recent lipidome profiling studies have revealed specific lipidome signatures that surpass HDL cholesterol levels in predicting major adverse cardiovascular events (MACE). Recent work highlights the dynamic nature of the HDL lipidome as a critical determinant of HDL particle functionality, governing particle surface fluidity, interactions with cellular membranes, and cholesterol efflux capacity.

The maturation and stability of the HDL core are regulated by LCAT and CETP. A key feature of HDL lipidomic disruption in patients with metabolic syndrome and coronary artery disease is the depletion of CEs and the concurrent enrichment of triglycerides within the hydrophobic core. This triglyceride accumulation, often driven by CETP, compromises the structural stability of the HDL particle, increasing its susceptibility to hydrolysis by hepatic and endothelial lipases. Such CETP-mediated lipid redistribution, coupled with impaired LCAT activity in metabolic disease, further impairs HDL maturation and promotes the formation of dysfunctional, small, dense HDL particles. Consequently, the resulting small, dense HDL particles exhibit a reduced circulatory half-life and a diminished capacity for cholesterol efflux [79]. Among the most clinically relevant lipidomic shifts is the alteration of sphingolipid species, particularly S1P. Under physiological conditions, HDL-bound S1P is a critical mediator of endothelial homeostasis, promoting NO production and maintaining the vascular barrier [80]. However, in the context of acute coronary syndrome, S1P levels in HDL are significantly reduced, while pro-apoptotic species such as ceramides are abundant. This alteration in sphingolipid composition not only compromises the anti-inflammatory signaling ability of HDL but also activates of the NLRP3 inflammasome in macrophages, thereby promoting the progression of atherosclerotic plaques [81]. Although phosphatidylcholine is the predominant phospholipid class, minor species such as plasmalogens and lysophosphatidylcholine (LPC) play disproportionate roles in HDL function. Plasmalogens serve as a primary defense mechanism against LDL oxidation. The loss of these antioxidant lipids, a common characteristic of the lipidomic profiles of individuals at high risk for CVDs, exacerbates oxidative stress within the subendothelial space. In contrast, an abnormal accumulation of LPC, often generated by phospholipase A2, can transform HDL into a proinflammatory vehicle that stimulates the expression of endothelial adhesion molecules [64]. Nevertheless, research on these low-abundance lipid fractions remains exploratory and fails to replicate across populations. Regional dietary variations and pharmacological interventions, particularly statin therapy, frequently confound the baseline lipid profile and obscure lipid remodeling dictated by the disease itself. Addressing these discrepancies in future precision lipidomics requires a shift toward large-scale, methodologically unified validation cohorts.

### 4.3. HDL as a Nanocarrier for Noncoding RNAs

HDL is a stable and efficient carrier of noncoding RNAs in the circulatory system [82]. miRNAs suppress gene expression through the posttranscriptional regulation of mRNA stability and translation. This process is primarily mediated by specific receptor-mediated pathways, enabling HDL to deliver miRNAs with precision to target cells, thereby playing a crucial role in intercellular communication and influencing downstream gene expression and cellular function [83,84]. miR-223 is a well-characterized HDL-associated miRNA whose horizontal transfer serves as a novel mode of intercellular communication. miR-223 is exported from myeloid cells and transferred to recipient vascular endothelial cells via SR-BI [85]. Incubating human coronary artery endothelial cells and human umbilical vein endothelial cells with native HDL significantly increases intracellular mature miR-223 levels by approximately 2-fold within 1 h, reaching approximately 5-fold and over 15-fold enrichment after 16 to 24 h, respectively. Tracking experiments reveal that primary and precursor forms of miR-223 are entirely undetectable in recipient endothelial cells. Neither the transcriptional inhibitor Actinomycin D nor the siRNA-mediated silencing of Dicer (the core miRNA-processing enzyme) can abrogate the intracellular rise in mature miR-223 following HDL exposure. Functionally, this delivered miR-223 exerts posttranscriptional gene silencing via canonical RNA interference pathways. Native HDL is dramatically superior to reconstituted HDL, lipid-poor ApoA-I, and small unilamellar vesicles at curbing endothelial inflammation. Pathological states profoundly alter HDL’s ability to deliver miRNAs. For instance, differences in HDL-miRNA profiles between healthy individuals and patients with familial hypercholesterolemia indicate functional changes in the disease context [86]. In ASCVDs, the HDL miRNA profile is remodeled, and these changes are driven by metabolic factors such as diet, obesity, and diabetes. Compared with miRNAs carried by other lipoproteins, HDL-associated miRNAs display greater specificity in terms of both composition and function [87]. Dysregulated long noncoding RNAs (lncRNAs) and circular RNAs (circRNAs) modulate HDL remodeling by competing endogenous RNA (ceRNA) networks. Some lncRNAs and circRNAs act as molecular sponges, sequestering miRNAs that target HDL biogenesis genes and altering cholesterol efflux, particle maturation, and lipidation. For instance, lncRNA KCNQ1OT1 promotes atherosclerosis by sponging miR-452-3p to repress ABCA1, impairing cholesterol efflux and accelerating HDL dysfunction [88]. It is increasingly recognized that CircRNAs associate with HDL and modulate its stability and proinflammatory potential by sequestering proatherogenic miRNAs [89].

## 5. Potential Pathogenic Mechanisms of dHDL in Cardiovascular Injury

### 5.1. Molecular Mechanisms of dHDL Biogenesis

**Figure 3:**
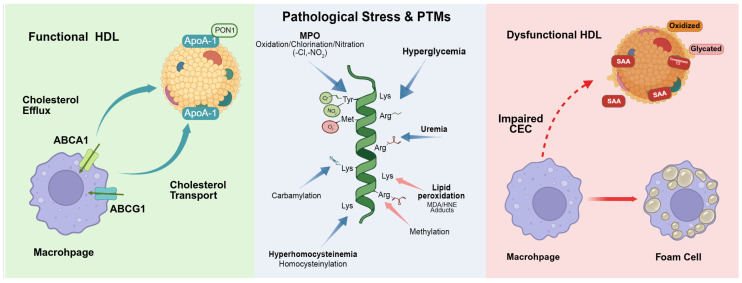
Site-specific posttranslational modifications of ApoA-I drive the structural and functional remodeling of HDL. Native ApoA-I undergoes PTMs under pathological stresses such as macrophage-derived MPO activity, hyperglycemia, and dyslipidemia. These site-specific modifications include MPO-mediated oxidation, chlorination, nitration (targeting Tyr/Met residues), glycation, carbamylation, and lipid peroxidation adduct formation. Abbreviations: PTMs, posttranslational modifications; PON1, paraoxonase 1; MDA, malondialdehyde; HNE, hydroxynonenal. Created in BioRender. serwe, F. (2026) https://BioRender.com/afbt3j0.

Under pathological conditions, HDL loses its vasculoprotective properties and is converted into a dysfunctional, pro-pathogenic mediator that contributes to structural and functional damage to the vascular wall via multiple heterogeneous molecular mechanisms. This remodeling is not simply a loss of function but rather the acquisition of deleterious properties that promote cardiovascular events. Notably, the phenotypic conversion of HDL to dHDL arises from a synergistic interplay of structural aberrations, site-specific posttranslational modifications (PTMs) on core ApoA-I, and the accumulation of pathological molecules, rather than isolated alterations in apolipoprotein composition alone (Figure 3) [17,34].

The normal physiological function of HDL is contingent upon its intact spherical structure. The primary manifestations of structural abnormalities in HDL include an imbalance in its subsets, aberrant particle morphology, and disrupted lipid–protein binding under pathological conditions. Specifically, remodeling often manifests as an imbalance among subfractions, frequently driving the miniaturization of dense HDL3 particles in metabolic disorders or, conversely, generating larger, structurally aberrant SAA-enriched particles during acute inflammation [90,91,92]. This change in shape reduces HDL’s ability to bind to receptors such as SR-BI and ABCA1, directly affecting reverse cholesterol transport.

Site-specific post-translational modifications (PTMs), particularly myeloperoxidase (MPO)-mediated oxidation within the subendothelial compartment, drive the conformational remodeling of ApoA-I into dysfunctional forms. MPO-catalyzed chlorination and nitration preferentially target critical tyrosine residues (notably Tyr192 and Tyr166) within the lipid-binding helices of ApoA-I, creating steric hindrance and altering local electrostatic surface potentials. Similarly, oxidation of Met86 and Met148 to methionine sulfoxide destabilizes the hydrophobic core of ApoA-I. Collectively, these site-specific modifications disrupt the lipid-binding interface and eliminate high-affinity interactions with ABCA1, thereby impairing cholesterol efflux capacity [93,94]. In chronic atherosclerosis, this site-specific oxidative modification blocks RCT and activates the NLRP3 inflammasome in subendothelial macrophages. The trapping of crystalline cholesterol and the reduction in PON1’s antioxidant defense result in a significant release of IL-1β and IL-18, creating an inflammatory setting that increases plaque vulnerability [95].

Nonenzymatic PTMs resulting from metabolic disturbances are another pathway that impairs HDL function. Nonenzymatic glycosylation induces the accumulation of advanced glycation end products (AGEs) on HDL particles. As glycemic dysregulation intensifies from early prediabetes to advanced type 2 diabetes (T2DM), AGE accumulation accelerates in a dose-dependent manner with rising ambient blood glucose levels [96]. This modification alters protein conformation. For example, in vitro studies have demonstrated that exposing HDL to elevated glucose levels induces ApoM dimerization. Additionally, treatment with methylglyoxal can modify the structure of ApoM, a change that can be mitigated by antiglycation agents [97]. The glycosylation of ApoA-I not only alters its structural integrity but may also affect its interactions with lipids and other proteins by modifying its conformation, thereby compromising the overall functional stability of HDL. Mechanistically, binding glycated lipoproteins to the AGE receptor on the endothelial cell membrane activates signaling pathways that reduce NO availability and increase oxidative and inflammatory stress, leading to endothelial dysfunction and atherosclerosis. Even in nondiabetic individuals with HbA1c below the diagnostic threshold, alterations in HDL occur during the subclinical phase of metabolic dysregulation [98,99]. Consequently, a hyperglycemic environment serves as a critical initial factor that precipitates abnormalities in HDL structure and function by facilitating the nonenzymatic glycosylation of HDL protein components.

Carbamylation represents another pivotal nonenzymatic posttranslational modification of HDL under pathological conditions, including atherosclerosis and uremia. Cyanate covalently and irreversibly modifies the N-terminal amino groups of the lysine residues on ApoA-I, alters its charge properties, and distorts its secondary and tertiary conformations, ultimately impairing its ApoA-I functionality [100,101,102]. Large amounts of carbamylated HDL are detected in human atherosclerotic plaques, where carbamylated proteins colocalize with MPO [100].

Carbonylation is a subtype of oxidative modification. Carbonyl compounds generated by lipid peroxidation or glycation covalently bind to lysine residues in ApoA-I, impairing its function and triggering endothelial injury. These carbonyl species include glyoxal, methylglyoxal, 4-hydroxynonenal, acrolein, malondialdehyde (MDA), and isolevuglandins (IsoLGs) [102,103]. MDA-modified HDL suppresses PON1 activity, activates protein kinase Cβ2, and inhibits endothelial nitric oxide synthase [70]. Notably, MDA selectively modifies ApoA-I lysine residues to disrupt RCT, a finding confirmed in human atherosclerotic plaques [104].

Similarly, homocysteinylation of HDL, typically driven by hyperhomocysteinemia, neutralizes the positive charge of lysine residues to compromise RCT [105]. This alteration further reduces PON1 activity, thereby diminishing HDL’s core antioxidant and anti-inflammatory properties, accelerating vascular remodeling, and driving matrix metalloproteinase-9-mediated extracellular matrix degradation within atherosclerotic plaques [106].

Aberrant accumulation of atypical molecules is another hallmark of the transformation of HDL into dHDL, and HDL functionality relies on the dynamic equilibrium of its lipid and protein components. Under pathological stress, the collapse of this dynamic balance drives a defining feature of dHDL biogenesis. This phenotypic alteration is characterized by the enrichment of ApoC-III, SAA, proinflammatory lipids, and abnormal proteases [107,108,109]. As a common uremic toxin in chronic kidney diseases, symmetric dimethylarginine (SDMA) is elevated in the circulation [110,111]. Clinical cohort evidence indicates that plasma SDMA levels are significantly elevated from the predialysis stage and exhibit a strong positive correlation with serum creatinine, accompanied by elevated levels of cytokines such as TNF-α and IL-6 [112,113]. This accumulation increases SDMA specifically within the HDL particles. SDMA-loaded HDL subsequently initiates endothelial dysfunction through Toll-like receptor 2 (TLR2) and matrix metalloproteinase-9 pathways. Elevated levels or aberrant deposition of these molecules within HDL not only disrupts the native protein–lipid interaction and structural stability of HDL but also directly endows HDL with proinflammatory properties, and abnormal expression of these molecules is strongly associated with increased CVD risk. Collectively, pathological microenvironments across major cardiovascular diseases and comorbidities drive the composition and PTMs of HDL, generating dysfunctional HDL with distinct pathogenic mechanisms that promote vascular injury and disease progression, as systematically summarized in Table 2.

### 5.2. Disease-Specific dHDL Remodeling and Pathogenic Mechanisms

#### 5.2.1. Abdominal Aortic Aneurysm

Alterations in HDL subfractions and the formation of dHDL are closely associated with pathological aortic wall remodeling in aneurysmal disease. Within abdominal aortic aneurysm lesions, MPO is highly expressed and catalyzes the oxidation of tyrosine and methionine residues on ApoA-I, disrupting its α-helical domain and impairing cholesterol efflux. Clinically, chronic inflammatory states are associated with enrichment of acute-phase reactants such as SAA and complement C3 within HDL particles. Recent studies further indicate that SAA-driven remodeling of HDL protein composition partly contributes to reduced cholesterol efflux capacity, presumably by displacing ApoA-I from the particle surface [117]. Excessive enzymatic activity within the aneurysm microenvironment has been shown to drive HDL lipid remodeling, characterized by a transition from lipid-poor nascent HDL to mature, cholesteryl ester-rich HDL. Elevated LCAT accelerates the maturation of nascent HDL through cholesterol esterification, whereas CETP facilitates the transfer of CEs to ApoB lipoproteins, thereby depleting the phospholipid-rich spherical HDL necessary for ABCG1-mediated efflux [118]. Increased proteolytic activity is critical in the aneurysm microenvironment. Neutrophil elastase cleaves ApoA-I, resulting in structurally abnormal HDL that is internalized and degraded by macrophages via SR-BI at approximately 3 times the rate of native HDL [119]. This process may also deplete HDL-bound α-1 antitrypsin, thereby potentially diminishing HDL’s antiproteolytic capacity. Additionally, evidence suggests that HDL can associate with matrix metalloproteinase-9, further impairing its functionality [120,121]. Beyond oxidation and proteolysis, MPO-derived cyanate carbamylates lysine residues on ApoA-I, disrupting receptor interactions and lipid binding, whereas carbamylated HDL initiates proinflammatory signaling pathways [100,122]. Glycation leads to the formation of AGEs, which destabilize HDL conformation and promote its clearance [123]. These modifications are thought to induce IgG autoantibodies against native and MDA-modified HDL, triggering immune-mediated HDL depletion and collectively ablating HDL’s vasculoprotective functions [124,125].

#### 5.2.2. Coronary Artery Disease

In patients diagnosed with CAD, the HDL inflammatory index reflects HDL’s capacity to suppress monocyte chemotaxis induced by oxidized oxLDL, appears to be significantly elevated compared with age- and sex-matched healthy controls, who exhibit comparable HDL-C concentrations [109,126,127]. This inflammatory microenvironment frequently correlates with the accumulation of SAA and complement C3. Concurrently, clinical studies suggest that MPO may exhibit heightened activity within vulnerable coronary plaques. In vitro evidence has indicated that MPO may facilitate the site-specific oxidation and chlorination of critical amino acid residues on ApoA-I, notably Tyr192 [128,129]. The resulting modified ApoA-I demonstrates a significantly diminished capacity to activate LCAT and fails to interact effectively with the macrophage ABCA1 and ABCG1 transporters, thereby impeding the rate-limiting step of RCT [130]. These structural, proteomic, and lipidomic alterations in HDL are believed to promote cholesterol accumulation in arterial macrophages, accelerate foam cell formation, and destabilize the plaque microenvironment, which may increase susceptibility to plaque rupture and promote the clinical progression of CAD. Beyond potentially contributing to acute ischemic events via plaque destabilization, the phenotypic transition to dHDL has also been implicated in compromising the efficacy of angiogenesis therapy. Under physiological conditions, HDL transports S1P, which activates the S1P1–WWP2–KLF5 signaling axis, thereby transcriptionally suppressing the long noncoding RNA HDRACA. This, in turn, may promote angiogenesis through the expression of proliferating cell nuclear antigen, thereby facilitating the formation of myocardial collateral circulation. In contrast, dHDL exhibits reduced S1P content, which impairs its capacity to suppress HDRACA. This mechanism may underlie the loss of proangiogenic activity observed in clinical CAD samples [131]. The loss of S1P also compromises eNOS activation, further exacerbating endothelial dysfunction and creating a state of therapeutic refractoriness. Clinical sample analyses have validated the pathological relevance of this signaling pathway, underscoring it as a novel therapeutic target for angiogenesis in CAD [132].

#### 5.2.3. Peripheral Artery Disease

Circulating oxidized HDL (ox-HDL), a prototypical dHDL subtype, is frequently associated with peripheral artery disease (PAD) severity and progression. Its levels tend to increase progressively with PAD severity and are often inversely correlated with vasoprotective small HDL particles. Notably, specific genetic variants may influence PAD pathogenesis by modulating HDL subfraction profiling and promoting oxidative modification of ApoA-I [133]. A large-scale prospective epidemiological study revealed that after adjusting for traditional cardiovascular risk factors, elevated triglyceride-related lipids (such as residual lipoprotein cholesterol) and reduced HDL-related lipids (such as HDL-C and ApoE-HDL) independently predicted the incidence of PAD [134]. Principal component analysis further corroborated that “triglyceride-related lipid components” and “HDL-related lipid components” independently contributed to the risk of PAD. These findings suggest that the core lipoprotein abnormalities linked to PAD development appear to be characterized by an increase in triglyceride-rich lipoproteins and a depletion of functional HDL, rather than a mere increase in low-density lipoprotein cholesterol (LDL-C). This transition from HDL to dHDL is accompanied by a loss of PON1 activity and a significant accumulation of ox-HDL, potentially resulting in a diminished capacity of HDL to neutralize oxidized lipids and inhibit inflammatory signals [133]. Previous studies have demonstrated that the anti-inflammatory ability of HDL3 subsets isolated from PAD patients is significantly impaired. However, in PAD patients with diabetes, HDL3 expression can even completely shift to a proinflammatory phenotype, which regularly upregulates endothelial cell adhesion molecules and promotes monocyte adhesion to the vascular endothelium [135].

#### 5.2.4. Cerebrovascular Diseases

dHDL may exert pathogenic effects on cerebrovascular disorders, extending its role in vascular pathology to the central nervous system. Accumulating evidence implicates dHDL as a key pathogenic mediator in cerebrovascular diseases such as stroke and vascular dementia, where it potentially contributes to cerebral atherosclerosis, disrupts blood–brain barrier integrity, and exacerbates neuroinflammation. Its pathogenic role is further underscored by clinical associations with reduced PON1 activity and impaired clearance of neurotoxic aggregates, linking systemic lipid dysfunction to cerebral vascular and cognitive impairment [136].

### 5.3. dHDL Biogenesis Driven by Metabolic and Uremic Microenvironment Dysregulation

#### 5.3.1. Diabetes Mellitus

The diabetic metabolic milieu has been strongly associated with dHDL biogenesis. In patients with insulin resistance or T2DM, HDL levels frequently undergo a qualitative transformation. This metabolic derangement initially tends to promote the formation of small, dense HDL particles characterized by triglyceride enrichment and cholesteryl ester depletion [137,138,139]. Notably, compared with patients with T2DM and normal triglyceride levels, patients with T2DM and concomitant hypertriglyceridemia often exhibit unexpected increases in CEC. This paradoxical elevation can be attributed to an expanded pool of triglyceride-rich lipoproteins, which serve as a lipid sink for CEs transferred from HDL via CETP. This transfer promotes the depletion of HDL core lipids and the dissociation of lipid-poor or lipid-free APOA-I, thereby enhancing overall CEC independent of genuine HDL functionality. However, this “false prosperity” in CECs may not reflect improved HDL function. Instead, diabetes appears to compromise key steps in the RCT pathway. Hyperglycemia-induced glycation of ApoA-I changes its shape and inhibits its ability to activate LCAT, thereby compromising the cholesterol esterification step of RCT. Recent studies have shown that ABCA1-mediated cholesterol efflux to small HDL particles may be diminished in patients with T2DM. These observations suggest that HDL function in diabetes should ideally be evaluated beyond total CEC, placing greater emphasis on its functional integrity and defects.

Chronic hyperglycemia induces the glycation of ApoA-I and related apolipoproteins and triggers oxidative modifications of lipids, apolipoproteins, and functional enzymes in HDL. These compositional changes alter the steric shape of ApoA-I, decrease its binding affinity to the lipoprotein surface, and accelerate the clearance of HDL particles, potentially leading to reduced HDL particles. Such glycation is considered a key molecular link between disturbed glucose metabolism and lipoprotein dysfunction. Clinical studies show that the half-life of glycated ApoA-I is significantly shorter, and the turnover rate is often inversely related to HbA1c levels, directly contributing to the impairment of functional HDL.

Beyond lipid and structural remodeling, diabetes inflammation drives a major proteomic shift that leads to dHDL formation. Increased levels of SAA and ApoC-III substitute the core functional protein ApoA-I and promote the transformation of HDL to dHDL with a proinflammatory phenotype. Concurrently, high oxidative stress in T2DM leads to excessive ROS production, which not only induces oxidative modifications to the protein and lipid components of HDL but is also thought to significantly inhibit the activities of PON1 and PAF-AH. Together, these changes lead to a fundamental reversal of HDL’s antioxidant and anti-inflammatory functions. Its ability to inhibit LDL oxidation, alleviate cellular oxidative stress, and suppress the expression of adhesion molecules, such as vascular cell adhesion molecule-1, in endothelial cells is severely impaired. In some cases, dHDL may even increase ROS production in endothelial cells, potentially transitioning them from protectors into pathogenic mediators.

A comparative analysis demonstrated that, despite preserved cholesterol efflux capacity, the antioxidant efficacy of HDL from diabetic patients is compromised compared with that of healthy controls [140]. This may explain why diabetic HDL tends to exhibit a selective, rather than synchronous, loss of protective properties. While cholesterol efflux capacity may be temporarily preserved through compensatory mechanisms, antioxidant, anti-inflammatory, and endothelial protective functions appear to be impaired at an early stage. This functional dissociation phenomenon, particularly evident in individuals with type 1 diabetes mellitus, who have normal HDL-C levels but globally defective function, underscores the critical importance of evaluating HDL quality rather than quantity in cardiovascular risk management for diabetes.

#### 5.3.2. Chronic Kidney Disease

Chronic kidney disease drives HDL remodeling, which is primarily orchestrated by the accumulation of protein-bound uremic toxins. Specifically, SDMA enrichment has been reported to transform HDL into a potent proinflammatory mediator. SDMA-modified dHDL has been shown to act as an endogenous ligand for TLR2 across different cell types. In monocytes and macrophages, this binding activates downstream cascades, potentially triggering a significant release of TNF-α and IL-6 [141,142]. SDMA-HDL appears to induce endothelial dysfunction by specifically targeting endothelial TLR2, thereby bypassing the canonical HDL receptor, SR-BI, and the TLR1/TLR6 complex. Hesse et al. reported that this TLR2-mediated endothelial damage is closely linked to the pathological degradation of the endothelial glycocalyx [113]. Clinically, circulating SDMA serves as a predictor of cardiovascular and all-cause mortality, a phenomenon that appears to be closely associated with its preferential accumulation in HDL particles and subsequent transformation into vasculotoxic entities [143].

## 6. Assessment of the Evolving Landscape of HDL

HDL evaluation has evolved from a static cholesterol count to high-resolution measurements of particle number, size distribution, and biological potency. Traditional clinical diagnostic methods depend on automated homogeneous assays [144]. While these assays provided the epidemiological foundation for the inverse association between HDL-C and cardiovascular risk, they offer only a static quantification of cholesterol mass. These approaches fail to account for the proteomic complexity, structural diversity, and dynamic functionality of HDL particles [145]. As a result, NMR spectroscopy has become integral to deep phenotyping, using the unique spectroscopic signatures of lipid methyl groups to perform detailed analyses of HDL particle concentrations and their distribution across specific size subfractions [146]. This physical characterization is often complemented by ion mobility analysis, which employs gas-phase electrophoretic mobility to accurately delineate the hydrodynamic radii of lipoprotein subspecies with exceptional reproducibility [147]. However, as the association between HDL-C levels and clinical outcomes has become clearer, the focus has shifted toward standard functional analyses [148,149,150,151]. CECs, the gold standard of HDL efficiency, reflect the ability of apoB-depleted serum to facilitate the removal of radiolabeled or fluorescently tagged cholesterol from J774 or THP-1 macrophages, primarily via the ABCA1 and ABCG1 pathways [152]. Its efficacy depends primarily on the intracellular cholesterol content of macrophages, the expression of ABCA1/ABCG1 transporters, and the lipid and protein composition of HDL, which acts as an extracellular receptor. Despite its ability to predict cardiovascular outcomes, the widespread implementation of CECs remains limited because they are time-consuming and low-throughput and require cultured cells [153]. To address the inflammatory transition of HDL particles, cell-free assays using fluorogenic probes, such as 2′,7′-dichlorodihydrofluorescein (DCFH), are used to quantify the HDL inflammatory index, thereby distinguishing antioxidant-rich homeostatic particles from pro-oxidant dysfunctional variants [154]. The frontier of HDL evaluation has transitioned from isolated biomarker analysis to high-throughput, mass spectrometry-based deep phenotyping, enabling comprehensive assessment of particle molecular stoichiometry. Recent advancements in LC-MS/MS have facilitated the identification of more than 90 distinct proteins within the HDL proteome, extending beyond basic quantification of ApoA-I [64]. This proteomic resolution revealed that the enrichment of specific proteins, such as ApoC-III and SAA, is a significant predictor of future cardiovascular events, effectively identifying high-risk individuals even when circulating HDL-C levels appear normal. To compare the performance characteristics and application scenarios of the HDL assessment approaches, we summarize the core attributes in Table 3.

Despite these methodological advances, several critical gaps prevent the translation of HDL functional and omics assays into routine clinical practice. There is no universally harmonized protocol for most functional HDL assays. CEC assays vary substantially across laboratories in cell source, cholesterol labeling method, incubation duration, and calculation formula, while mass spectrometry-based proteomic and lipidomic assays lack unified sample preparation workflows and quantitative standards. Protocol heterogeneity leads to poor concordance of quantitative results between research centers, limiting comparison of data across study cohorts and hindering further validation. To date, most published associations are derived from single-center observation; there are no prospectively validated, universally accepted clinical threshold values for HDL functional parameters or proteomic signatures.

These methodological constraints represent a bottleneck for the clinical translation of HDL functional assessment. By integrating structural signatures with kinetic functional data, researchers have developed multidimensional assessments for evaluating HDL biology. A seminal study by Rohatgi et al. demonstrated that compared with static cholesterol measurements, CEC is a superior predictor of incident coronary heart disease. These findings have been further corroborated by machine learning models that integrate proteomic stoichiometry with lipidomic profiles to derive an index reflecting HDL-related inflammation [29]. This integrated approach provides a far more robust predictive framework for atherosclerotic progression and plaque instability by capturing the particle’s dynamic properties rather than its mere circulating mass. Consequently, these multidimensional assessments are advancing the evolution toward precision lipidology by enabling the identification of specific pathological HDL clusters that can be targeted through personalized therapeutic interventions, thereby bridging the gap between molecular discovery and clinical vascular practice.

## 7. Therapeutic Implications and Challenges

Despite decoding the molecular features of dHDL, translating these findings into improved cardiovascular outcomes remains a challenge [43]. One question is whether the transformation of native HDL to dHDL is a stochastic process resulting from cumulative damage or a regulated event initiated by a discrete “master switch”. Identifying such a pivotal node—potentially a proteomic cluster involving SAA/MPO or a lipidomic signature such as the S1P-HDRACA axis—could revolutionize our ability to intervene early. Second, the “HDL-C paradox” has rendered static mass measurements insufficient for modern risk stratification. While CEC outperforms static HDL-C measurements in predicting cardiovascular risk, no standardized, high-throughput, clinically deployable assay for HDL functional assessment has been adopted [153,155]. Integrating functional HDL testing into clinical workflows would require automated platforms with rigorous interlaboratory harmonization and reference ranges and would demonstrate additive prognostic value beyond existing biomarkers. To address these unmet needs, future initiatives must prioritize establishing international consensus on sample preprocessing and detection workflows while advancing cell-free technologies, such as fluorescent probe-based CEC assays or oxidized ApoA-I-targeted immunological detection, to ensure scalability. These advancements should be paired with large-scale prospective trials across diverse clinical phenotypes, including diabetes, chronic kidney disease, and autoimmune disorders, to define precise prognostic cutoff values. Correlating these functional indices with advanced atherosclerotic imaging, such as coronary computed tomography angiography and intravascular ultrasound, along with major adverse cardiovascular events, is essential to establish their validity as robust surrogate endpoints in cardiovascular research and therapeutic monitoring. Third, development should focus on targeted therapeutic strategies that neutralize pathological molecules within dHDL or reinstate essential ligands specifically at the site of vascular injury. The history of CETP inhibitors and niacin serves as a cautionary tale: simply raising HDL-C levels does not equate to vascular protection. Therapeutic strategies that broadly alter HDL composition or metabolism risk unintended off-target effects. We have evaluated the clinical evidence and the functional impacts on HDL of four major classes of HDL-targeted and HDL-related therapies.

CETP inhibitors are the most extensively studied HDL-raising agents, but their clinical development has been largely marked by neutral or adverse cardiovascular outcomes [156,157]. This therapeutic failure may be explained by structural and functional abnormalities in remodeled HDL particles [158]. Both Torcetrapib and Evacetrapib failed to reduce major adverse cardiovascular events despite elevating circulating HDL-C levels [13,151]. Post hoc analyses revealed that these agents structurally disrupted the HDL proteome by enriching particles with ApoC-III. Anacetrapib achieved a modest clinical benefit, which was probably due to a reduction in LDL-C rather than an elevation in HDL-C. Structurally, anacetrapib induces the accumulation of HDL2 subfractions [159]. Similarly, dalcetrapib expands HDL mass but stimulates the generation of ApoC-III-enriched variants, resulting in no overall cardiovascular benefit [149].

ApoA-I-targeted strategies aim to restore or simulate native HDL functional biology. CSL112, a formulation of wild-type human ApoA-I complexed with phospholipids, significantly increased ApoA-I levels and peripheral CEC in the AEGIS-I trial [160]. However, it did not reduce the 90-day MACE risk. The short synthetic amphipathic α-helical peptides designed to mimic ApoA-I failed to demonstrate significant regression of coronary plaque in clinical trials [161,162]. Upregulating endogenous ApoA-I synthesis via inhibition of bromodomain and extra-terminal (BET) proteins represents an epigenetic shift. However, the phase 3 BETonMACE trial did not meet its primary endpoint of reducing MACE [163].

Chronic inflammation promotes the transition of HDL into a pro-pathogenic mediator, thereby dampening upstream inflammatory cascades and representing an indirect method of functional rescue. For instance, Janus kinase (JAK) inhibitors used to treat rheumatoid arthritis have been shown to restore HDL subclass distribution by increasing HDL2 particle levels. These improvements correlate with reductions in C-reactive protein levels and with the restoration of the inherent antioxidant and anti-inflammatory activities of HDL [164].

Glucagon-like peptide-1 receptor agonists (GLP-1 RAs) and sodium–glucose cotransporter 2 inhibitors (SGLT2i) are established cardioprotective agents in metabolic disease, and their impacts on HDL function represent an area of active investigation. Clinical studies have demonstrated that GLP-1 RAs restore HDL function in patients with T2DM, independent of changes in HDL-C levels [165]. However, few clinical cohorts have characterized the effects of SGLT2i on HDL protein glycation dynamics, molecular stoichiometry, or subspecies distribution, despite the well-established cardioprotective and metabolic benefits of SGLT2i [166].

Collectively, these therapeutic strategies offer critical lessons for HDL-based drug development. The failure of CETP inhibitors and niacin to reduce cardiovascular events despite HDL-C elevation has refuted the “HDL-C-raising” hypothesis, establishing that HDL function, rather than cholesterol mass, is the therapeutic target. The neutral results from CSL112 and ApoA-I mimetics highlight the profound challenge of recapitulating HDL’s pleiotropic functions with a single agent, suggesting that successful intervention may require combination strategies that simultaneously address particle quantity, composition, and functional integrity. The indirect approaches, such as anti-inflammatory therapies and metabolic agents, in restoring HDL function underscore the intimate link between HDL functionality and the systemic metabolic milieu, suggesting that restoring HDL function may be achieved by treating the underlying disease state.

Targeting diverse pathological mechanisms requires etiology-specific therapies tailored to dysfunctional HDL subtypes. For instance, small molecules neutralizing AGEs could reverse diabetic dyslipidemia, while localized, inflammation-driven HDL remodeling requires targeted immunomodulation. Delivering these interventions precisely to atherosclerotic plaques can be achieved using nanotechnology and bioengineered lipoproteins, which facilitate site-specific accumulation and controlled release of functional cargo such as ApoA-I or S1P [167]. Patient stratification through multiomics profiling will be essential for identifying cohorts most likely to benefit from this molecular restoration, thereby enabling the integration of these modulators into standard cardiovascular care. Note that some HDL-related concepts discussed in this paper are primarily supported by mechanistic or preclinical data; their causal relevance and translational applicability in humans warrant further investigation in clinical cohort studies.

## 8. Conclusions

To better understand and manage HDL-associated CVDs, research should move beyond static HDL-C measurements. Rather, a generalized mechanistic approach should be adopted to understand the structural and functional heterogeneity of HDL and its dynamical transformation into dHDL in pathological environments. To overcome the limitations of the “HDL-C paradox”, clinical evaluation should shift to integrated multidimensional profiling. Future mechanistic studies should leverage stable isotope-based kinetics and spatial multiomics to trace the precise chronological remodeling of the HDL proteome and lipidome under specific conditions, such as inflammation or uremic stress. Emerging therapeutic approaches that restore ApoA-I and PON1, neutralize dHDL-enriched pathological molecules, or modulate S1P signaling are promising for reversing HDL dysfunction and restoring vascular protection. The adoption of a mechanism-driven research paradigm, coupled with stratified clinical assessment of HDL from structural and functional characterization to the identification of dHDL-specific pathogenic targets, may facilitate the development of genuinely personalized lipid-lowering and vascular-protective therapies. This approach is poised to advance the overarching goal of enhancing cardiovascular clinical outcomes and alleviating the global burden of CVDs and comorbidities.

## Figures and Tables

**Table 2:** Pathogenic mechanisms of dHDL in cardiovascular diseases and comorbidities.

Disease	Pathological Microenvironment	HDL Modifications	Potential Pathogenic Mechanisms of dHDL	Ref
Abdominal Aortic Aneurysm	High proteolytic activity, adventitial inflammation, MPO overexpression	Neutrophil elastase-mediated ApoA-I cleavage; MPO-driven oxidation/carbamylation; MDA adduction; α1-antitrypsin depletion; AGEs formation	Impaired cholesterol efflux; compromised anti-proteolytic activity; proinflammatory signaling; HDL depletion	[92]
Coronary Artery Disease	Plaque inflammation, localized oxidative stress, MPO hyperactivation	MPO-mediated oxidation & chlorination (Tyr192); SAA & C3 enrichment; S1P depletion	Impaired RCT; blunted eNOS activation; disrupted S1P-HDRACA pro-angiogenic axis	[17]
Peripheral Artery Disease	Systemic atherosclerosis, hypertriglyceridemia, chronic inflammation	Accumulation of ox-HDL; depletion of small functional HDL, PON1 activity loss	Compromised anti-inflammatory/antioxidant capacity; upregulated endothelial adhesion molecules; enhanced monocyte adhesion	[114]
Cerebrovascular Diseases	Cerebral atherosclerosis, neuroinflammation	Reduced PON1 activity; systemic proteomic remodeling	Suppressed fibrinolytic activity via PAI-1; impaired protein C pathway; promoted SR-BI-dependent endothelial apoptosis; blunted eNOS/NO axis	[47]
Diabetes Mellitus	Chronic hyperglycemia, insulin resistance, oxidative stress	AGEs formation; TG enrichment & CE depletion; SAA/ApoC-III enrichment; PON1 inactivation	Impaired LCAT activation & RCT; clearance and functional uncoupling of HDL	[115]
Chronic Kidney Disease	Uremic toxicity, chronic inflammation	SDMA enrichment; cyanate-mediated carbamylation	TLR2-mediated proinflammatory signaling (TNF-α/IL-6 release); endothelial glycocalyx degradation; endothelial dysfunction	[116]

**Table 3:** Comparative summary of HDL assessment.

Assay	Mechanisms	Method	Advantages	Challenges
Cholesterol efflux capacity	• Measures the ability of HDL to accept cholesterol from macrophages loaded with radiolabeled or fluorescent cholesterol.	• Cell-Based Assay	• Widely recognized gold standard. • Independently associated with CVD risk • Assesses the key step of RCT	• Laborious and time-consuming (48–72 h) • Requires cell culture facilities and expertise • Poor interlaboratory standardization • High cost
	• Measures the exchange or binding of fluorescently labeled phospholipids or cholesterol to HDL, without live cells.	• Cell-Free Assay	• Easy to perform and automated • Fast and high-throughput • Good reproducibility	• Requires more systematic clinical validation and standardized data analysis • May not fully capture the complex biological processes.
Antioxidant function	• Employs DCFH-DA to measure the capacity of HDL to inhibit LDL oxidation.	• DCF-Based Fluorescence Assay	• Simple and cost-effective • High-throughput potential	• Nonspecific oxidation • Variable specificity and sensitivity
	• Measures the ability of HDL to reduce the formation of oxidized phospholipids	• Oxidized Phospholipid Assay	• Assesses HDL’s capacity to detoxify pro-inflammatory oxidized lipids	• Lack of normal reference values
Enzyme Activity Assay	• Measures the capacity of HDL-associated PON1 to hydrolyze oxidized lipids	• HDL Paraoxonase 1 Activity	• Independently associated with CVD risk and severity • Surrogate estimated of HDL antioxidant capacity • Easy to perform and cost-effective • High throughput and feasible for automation	• Lack of standardization • Lack of normal reference values
Particle Characterization	• Quantifies HDL particle number, size, and subclass distribution from the lipid methyl signals in plasma	• Nuclear Magnetic Resonance Spectroscopy	• High-throughput • Reproducible • Requires no prior isolation of HDL • HDL-P and certain subclasses (e.g., small HDL-P) can predict CVD risk better than HDL-C	• Different cutoff values for HDL-P size classification across studies • Expensive equipment and specialized expertise required • Does not assess protein or lipid composition.
Proteomic/Lipidomic Profiling	• Quantifies HDL protein and lipid composition at the molecular level	• Liquid Chromatography-Tandem Mass Spectrometry	• High sensitivity & specificity • Identifies and quantifies rare modifications • Provides molecular characterization	• Complex sample preparation • Lower throughput • High cost • Lack of standardization

## Data Availability

No new data were created or analyzed in this study. Data sharing is not applicable to this article.

## Abbreviations

The following abbreviations are used in this manuscript:

ABCA1	ATP-binding cassette transporter A1
ABCG1	ATP-binding cassette transporter G1
AGEs	Advanced glycation end products
ApoA1	Apolipoprotein A-I
ApoB	Apolipoprotein B
ApoC-III	Apolipoprotein C-III
ApoE	Apolipoprotein E
ASCVD	Atherosclerotic Cardiovascular Disease
CAD	Coronary Artery Disease
CE	Cholesteryl Esters
CEC	Cholesterol efflux capacity
CETP	Cholesteryl Ester Transfer Protein
CVD	Cardiovascular Disease
DHCR24	3-β-Hydroxysteroid-Δ24 reductase
dHDL	Dysfunctional high-density lipoprotein
eNOS	Endothelial nitric oxide synthase
HDL	High-Density Lipoprotein
HDL-C	High-density lipoprotein cholesterol
IL-6	Interleukin-6
LCAT	Lecithin Cholesterol Acyltransferase
LDL	Low-Density Lipoprotein
LDL-C	Low-density lipoprotein cholesterol
MACE	Major adverse cardiovascular events
MDA	Malondialdehyde
NLRP3	NOD-, LRR- and Pyrin Domain-containing Protein 3
NO	Nitric oxide
oxLDL	Oxidized Low-Density Lipoprotein
PAF-AH	Platelet-activating factor acetylhydrolase
PAD	Peripheral Artery Disease
PI3K	Phosphatidylinositol 3-kinase
PLTP	Phospholipid Transfer Protein
PON1	Paraoxonase 1
PTMs	Posttranslational Modifications
RCT	Reverse Cholesterol Transport
ROS	Reactive Oxygen Species
SAA	Serum Amyloid A
SDMA	Symmetric Dimethylarginine
S1P	Sphingosine-1-Phosphate
SR-BI	Scavenger receptor class B type I
T2DM	Type 2 Diabetes Mellitus
TG	Triglyceride
TLR2	Toll-like Receptor 2
TNF-α	Tumor Necrosis Factor-α
WWP2	WW Domain Containing E3 Ubiquitin Protein Ligase 2
